# Amino acid profiles in the tissue and serum of patients with liver cancer

**DOI:** 10.1515/med-2022-0589

**Published:** 2022-11-18

**Authors:** Da-Hua Liu, Gui-Min Wen, Chang-Liang Song, Li-Jun Ji, Pu Xia

**Affiliations:** Biological Anthropology Institute, Jinzhou Medical University, Jinzhou, Liaoning, P.R. China; Department of Basic Nursing, College of Nursing, Jinzhou Medical University, Jinzhou, Liaoning, P.R. China; Department of Radiotherapy, Center Hospital of Handan, Handan, Hebei, P.R. China; Office of Library, Jinzhou Medical University, Jinzhou, Liaoning, P.R. China

**Keywords:** amino acid, liver cancer, liquid biopsy, prognosis, metabolism

## Abstract

Most patients with liver cancer were found late and lost the chance of surgery. Liquid biopsy can monitor the risk of tumor recurrence and metastasis, quickly evaluate the curative effect of tumor treatment, and is conducive to early screening and auxiliary diagnosis of high-risk groups. Amino acid (AA) profiling has been used to the diagnosis and the prognosis for cancers. However, little was known about the profiles of AA of liver cancer. In this study, we used tRNA in Cancer database to analyze the AA levels in liver cancer tissues. Blood samples of patients with liver cancer were collected and analyzed using the automatic AA analyzer. We found that valine, isoleucine, and leucine were decreased significantly both in the plasma and the tumor tissues of patients with liver cancer. However, upregulation of methionine was observed in tissues and plasma of patients with liver cancer. Interestingly, tyrosine, and phenylalanine were decreased in tumor tissue but increased in the plasma of patients with liver cancer. This is the first report provided an overview of AA profile in both plasma and tissue for patients with liver cancer. AA levels can be used as diagnostic and prognostic markers of patients with liver cancer.

## 1 Introduction

Cancer is a kind of metabolic disease [1]. Amino acids (AAs) are the basic units of proteins and regulators of metabolism [2]. A range of human diseases are marked by AAs, including cancer [3]. Compared to normal cells, cancer cells require more AA to synthesize proteins and nucleic acids [4]. AA profiling has been used to the diagnosis and the prognosis for lung [5], ovarian [6], head and neck [7], and gastric cancers [8]. It depends on cancer cell types and extracellular components and reflects the characteristics of cell proliferation and differentiation [9,10]. Liver is the main organ of AA metabolism [11]. The changes in AA spectrum are different in different degrees of liver damage [12]. Because of the serious damage of liver cells in patients with liver cancer, its metabolic function is affected [13]. The etiology and exact molecular mechanism of primary liver cancer are not fully understood. It is believed that its pathogenesis is a complex process with multiple factors and steps and is affected by both environmental and dietary factors [14,15,16,17]. Most patients with primary liver cancer are in the middle and late stages when they have obvious symptoms, so reliable tumor markers are needed for the early detection and diagnosis of liver cancer. Most of previous studies on hepatocellular carcinoma (HCC) metabolism have focused on glucose metabolism [18], lipid metabolism [19], and fatty acid metabolism [20]. AAs are involved in the synthesis of nucleic acids and proteins, which play an important role in the nutrition and treatment of patients with liver cancer [21]. However, until now, little was known about the profiles of AA of liver cancer. So, AA quantification is needed for the patients with liver cancer.

This study is focused on the AAs that can be used as biomarkers for liver cancer detection and prediction. We used the TCGA database to analyze the AA profiles in liver cancer and the roles of these AAs in the prognosis of the patients. In addition, we compared the plasma AA levels of patients with liver cancer with healthy volunteers.

## 2 Materials and methods

### 2.1 Bioinformatics analysis

tRNA in Cancer (tRic) is a comprehensive database for tRNAs in cancer (https://hanlab.uth.edu/tRic/" https://hanlab.uth.edu/tRic/) [22]. tRic has four functional modules: tRNA level, codon level, AA level, and codon usage [22]. We explored the AA frequency in liver hepatocellular carcinoma (LIHC) tissues among different stages, subtypes, and grade groups. Univariate Cox model was used to test the correlation of the AA frequency with patient survival. Difference in AA frequency was compared by Students’ *t*-test and *P* < 0.05 was considered as statically significant.

### 2.2 Blood samples

Blood samples of patients with Liver cancer (*n* = 82) were obtained from the Department of General Surgery, the Center Hospital of Handan, between September 2017 and June 2020. The patients did not receive chemotherapy or radiotherapy prior to blood sample collection. Control blood samples were obtained from 30 healthy individuals with similar age and daily lifestyle as the patients. This study was conducted according to the Helsinki Declaration of 1975 and approved by the Ethics Committee of Liaoning Medical University.

### 2.3 Detection of AAs

Blood sample (2 mL) was centrifuged at 3,000 rpm for 10 min, and plasma was separated. Sulfosalicylic acid (8%) and plasma were mixed at the ratio of 1:2 (V/V) and centrifuged at 15,000 rpm for 30 min. The supernatant was filtered and degassed by 0.45 μm microporous membrane. The samples were analyzed using the automatic AA analyzer (HITACHI L-8900, Japan).

### 2.4 Statistical analysis

GraphPad Prism 5 software (GraphPad Software Inc., San Diego, CA, USA) was used to analyze all experimental data and clinical data. One-way analysis of variance was used for the inter-group comparison. *P* < 0.05 was statistically significant.

## 3 Results

### 3.1 AA frequency and roles in LIHC tissues

**Figure 1:**
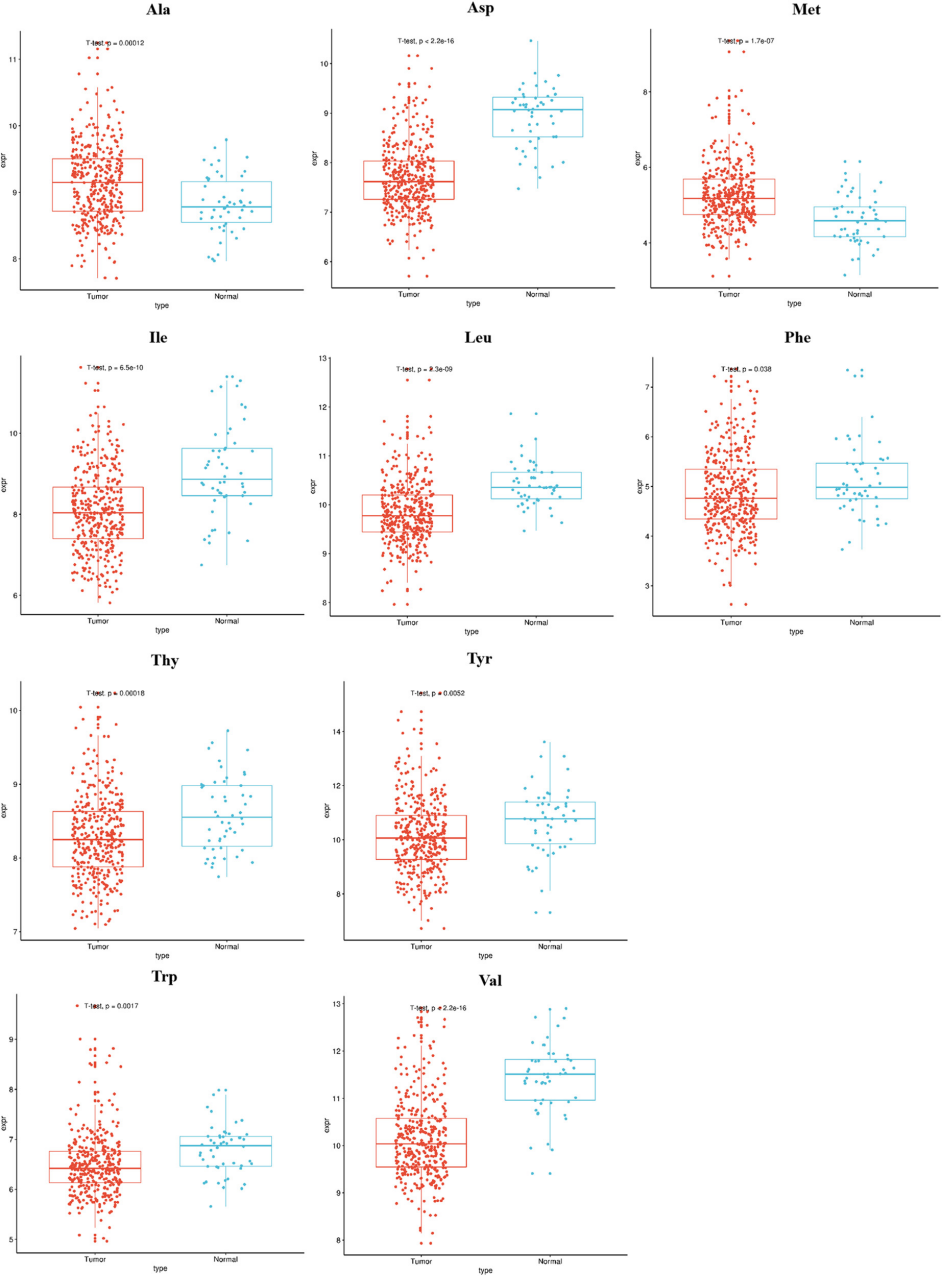
AA levels in tumor tissues and matched normal tissues of patients with liver cancer.

**Figure 2:**
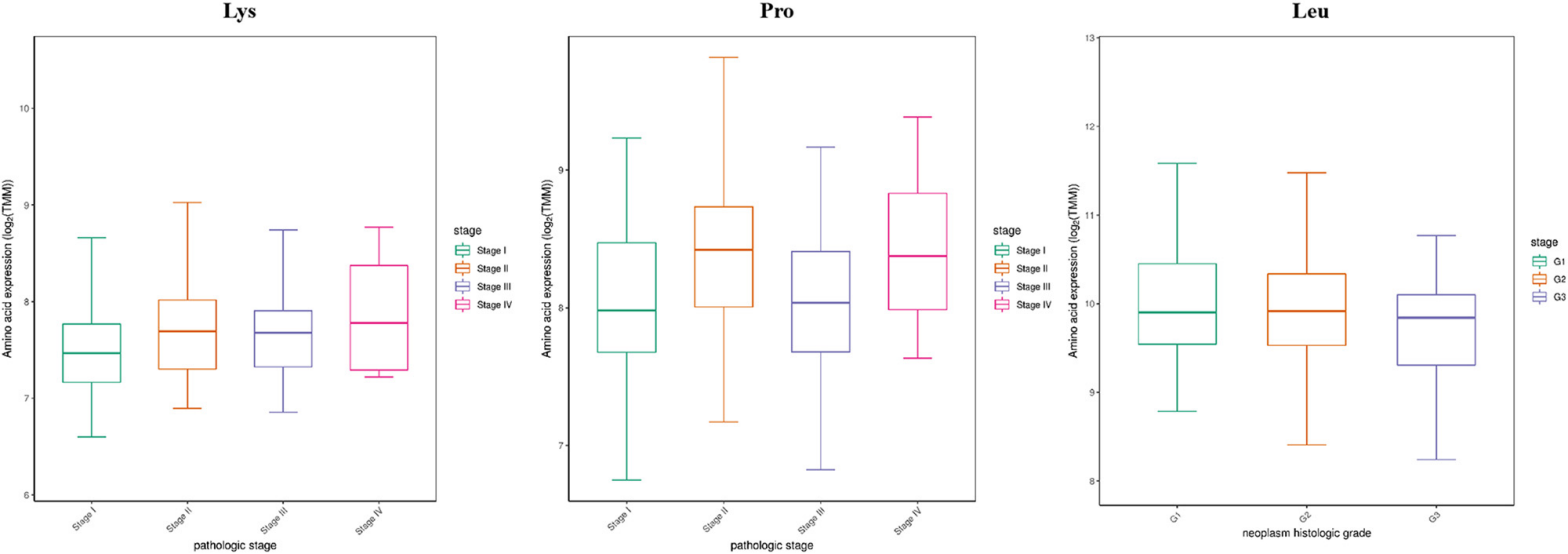
Differentially expressed AAs among different tumor stages of patients with liver cancer.

**Figure 3:**
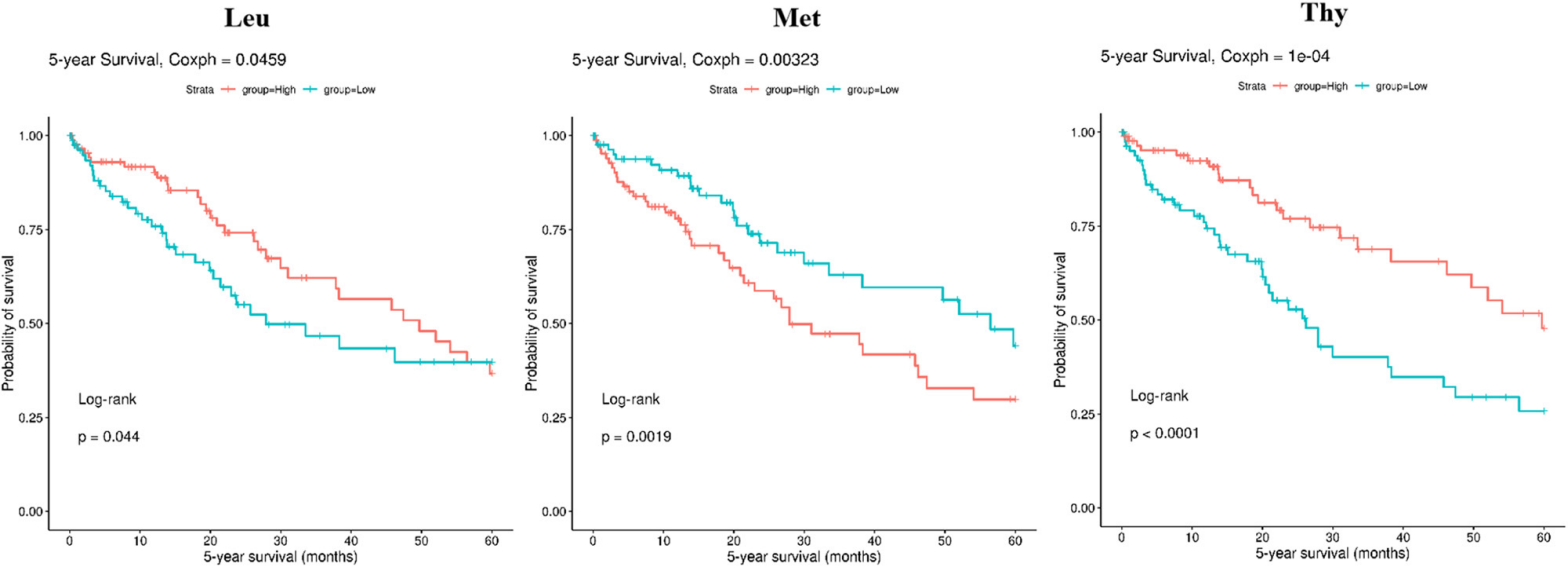
Differentially expressed AAs among different tumor grades of patients with liver cancer.

The levels of alanine (Ala) and methionine (Met) were higher in LIHC tissues than in matched normal tissues (*P* < 0.05, Figure 1). Leucine (Leu), isoleucine (Ile), aspartate (Asp), threonine (Thr), tyrosine (Tyr), tryptophane (Trp), valine (Val), and phenylalanine (Phe) were highly expressed in normal tissues (*P* < 0.05, Figure 1). Stage I LIHC tissues have lower concentration of Lys and Pro than that in stage II–IV (*P* < 0.05, Figure 2). Higher Leu level was observed in grade 1 LIHC tissues compared with grade 2–3 LIHC tissues (*P* < 0.05, Figure 2). High levels of Leu and Thy indicated a good outcome of patients with LIHC (*P* < 0.05, Figure 3). However, the patients with low level of Met lived longer than the patients with high level (*P* < 0.05, Figure 3). Other AAs did not have significant differences in LIHC tissues compared to matched normal tissues (data not shown).

### 3.2 Serum AA profiles in patients with LIHC

The differences of serum AA between patients with LIHC and healthy volunteers are summarized in Table 1. Tyr, Met, and Phe in the serum of patients with LIHC were significantly higher than those in the healthy volunteers (*P* < 0.05). The levels of Val, Leu, Ile, arginine (Arg), and the ratio of branched chain amino acids (BCAAs) to aromatic amino acids (AAAs) in the patients with LIHC were significantly lower than those in the healthy volunteers (*P* < 0.05). Other AAs in serum, such as taurine (Tau), Asp, serine (Ser), glycine (Gly), Ala, lysine (Lys), histidine (His), and Trp, did not have differences in the two groups (*P* > 0.05).

**Table 1:** Serum AA profiles in patients with LIHC and healthy individuals ( \bar{x}\pm s] , μmol/L)

AAs	Patients with LIHC (82)	Healthy controls (30)
Val	**203.34 ± 34.56**	**261.29 ± 35.78**
Ile	**38.17 ± 15.45**	**67.87 ± 20.23**
Leu	**87.56 ± 18.38**	**124.42 ± 24.67**
Tyr	**95.56 ± 21.84**	**63.73 ± 17.77**
Phe	**125.61 ± 25.09**	**86.52 ± 20.40**
Arg	**40.27 ± 15.73**	**85.35 ± 22.34**
Met	**44.56 ± 13.34**	**26.27 ± 14.85**
Tau	90.36 ± 35.68	92.34 ± 30.46
Asp	14.35 ± 3.55	14.56 ± 3.67
Ser	135.73 ± 24.53	143.38 ± 22.37
Gly	256.42 ± 78.86	265.94 ± 74.53
Ala	409.36 ± 45.55	407.78 ± 43.49
Lys	160.76 ± 35.12	162.47 ± 36.62
His	77.24 ± 20.20	80.45 ± 18.85
Trp	50.79 ± 14.56	50.86 ± 15.87
**BCAA/AAA**	**1.45 ± 0.53**	**2.89 ± 0.71**

Branched chain amino acids: BCAA.

The AAs with black have statistically significance (*P* < 0.05).

## 4 Discussion

AAs are important factors for primary liver cancer cell, especially proliferation and metabolism [23]. The uptake rate of AAs by liver cancer cells is significantly accelerated [10]. Previous studies have demonstrated that a variety of AAs are involved in the occurrence and development of tumors [5,6,7,8]. Compared to healthy volunteers, serum AAs of the patients with liver cancer have changed, such as decreased Val, Ile, and Leu [24]. As we known, essential AA cannot be synthesized in the body and must be supplied by protein in food [25]. In this study, we analyzed AA levels in patients with liver cancer using the TCGA database and hope that our results can provide a basis for nutritional support for patients with liver cancer.

In our study, we found that Val, Ile, and Leu were decreased significantly both in the plasma and the tumor tissues of patients with liver cancer. Patients with liver cancer usually suffered hyperinsulinemia at the same time [26]. It promotes Val, Ile, and Leu into tissues and results in the decrease in them in serum of patients with liver cancer [26]. Val, Ile, and Leu can inhibit the formation and recurrence of HCC and reduces the risk for HCC [27]. In the study of Wu et al. [28], they confirmed that serum Val and Leu levels were lower in HCC than that in chronic hepatitis B. All the data in serum and tissue suggested that the ability of the protection of Val, Ile, and Leu decreased during HCC formation. Upregulation of Met was observed in tissues and plasma of patients with liver cancer. Met, a sulfur-containing essential AA, is a restriction factor for the synthesis of DNA and RNA in cancer cells [29]. Cancer cells need to consume a lot of Met for proliferation [23]. Therefore, the concentration of Met in patients with liver cancer is higher than that in healthy volunteers. Regulation of Met has been used as a therapy for patients with cancer [30]. We also found that Tyr and Phe were decreased in tumor tissue but increased in the plasma of patients with liver cancer. Previous studies also confirmed that Phe in the peripheral blood was higher in patients with HCC compared to that in patients with liver cirrhosis [28,31,32]. Increased serum Phe level indicated severe impaired liver function during HCC development [28,31,32]. Dietary restriction of Tyr and Phe in mice model decreased melanoma tumor growth rate compared to mice fed a normal diet [33]. In addition, liver is the main metabolic site of AAAs [34]. Due to the serious damage of liver cells in patients with liver cancer, its AAA metabolic capacity is declined [35]. So, the ratio of BCAA/AAA is significantly decreased.

In conclusion, the AA level of patients with cancer changed greatly compared to healthy volunteers. AAs also can be used as diagnostic and prognostic markers for cancers. This is the first report provided an overview of AA profile in both plasma and tissue for patients with liver cancer. AA levels can reflect the damage degree of liver cells and indicate the outcome of patients with liver cancer. Therefore, the detection of AAs in plasma has great clinical significance for patients with liver cancer.
